# Letter to the Editor: 'Heart failure with preserved ejection fraction (HFpEF): Diagnosis and management for the general physician'

**DOI:** 10.1016/j.clinme.2026.100627

**Published:** 2026-07-19

**Authors:** Maria Giulia Bellicini

**Affiliations:** Cardiology Institute, Spedali Civili di Brescia, Brescia, Italy

I read with great interest the comprehensive review on the contemporary diagnosis and management of heart failure with preserved ejection fraction (HFpEF) by Zakeri and Nabeebaccus.^1^ The authors provide a practical framework for non-specialists and appropriately emphasise three key concepts: HFpEF is a heterogeneous syndrome, elevated left ventricular filling pressures (particularly during exertion) represent its central haemodynamic abnormality and, importantly, exclusion of HFpEF mimics is a critical step in the diagnostic process.

We believe that this latter concept deserves even greater emphasis because it may have broader implications for how HFpEF is conceptualised in clinical practice. Throughout the review, the pathophysiological description of HFpEF is dominated by impaired cardiovascular reserve, chronotropic incompetence, exercise intolerance, and elevation of filling pressures during exercise. Likewise, the authors distinguish patients with overt congestion from those presenting predominantly with exertional symptoms without clear fluid overload, suggesting that the latter may represent earlier or less overt disease. This physiological framework portrays HFpEF primarily as a syndrome of exercise intolerance rather than one intrinsically characterised by recurrent episodes of acute pulmonary oedema.^2^

If this interpretation is accepted, the role of diagnostic mimics becomes particularly important. Significant valvular heart disease, infiltrative cardiomyopathies, precapillary pulmonary hypertensione are well-recognised causes of acute heart failure despite preserved ejection fraction. Consequently, an important unresolved question is how many patients currently labelled as having acute decompensated HFpEF would continue to fulfil this diagnosis after systematic exclusion of these alternative structural conditions. Real-world echocardiographic studies suggest that HFpEF mimics account for a substantial proportion of hospitalisations with preserved ejection fraction, raising the possibility that the prevalence of guideline-defined HFpEF presenting with acute congestion may be lower than generally assumed.^3^

Notably, the dynamic nature of functional valvular regurgitation may provide a possiblie explanation for at least some acute presentantion. Dynamic structural abnormalities, particularly atrial functional mitral and tricuspid regurgitation, may fluctuate considerably. Preliminary findings from the above-mentioned cohort further support this dynamic behaviour, as severe atrial functional valvular regurgitation frequently regressed at discharge or during follow-up.^3^ This mechanism may also help explain why atrial fibrillation represents one of the strongest and most consistent clinical features across patients currently classified as HFpEF, as atrial dysfunction and atrial functional mitral and tricuspid regurgitation may constitute common drivers of acute decompensation in a subset of these patients. Rather than representing contradictory entities, these mechanisms may coexist and account for part of the heterogeneity currently encompassed by the HFpEF label.

We therefore suggest that future diagnostic algorithms should not simply recommend exclusion of HFpEF mimics, but explicitly position this step hierarchically before assigning the diagnosis of HFpEF, particularly in patients presenting with acute heart failure and preserved ejection fraction. Such an approach may improve diagnostic specificity, facilitate identification of treatable structural disease, and contribute to a more homogeneous definition of HFpEF for both clinical practice and future therapeutic trials.

## CRediT authorship contribution statement

**Maria Giulia Bellicini:** Conceptualization, Writing – original draft, Writing – review & editing.

## Funding

This research did not receive any specific grant from funding agencies in the public, commercial, or not-for-profit sectors.

## Declaration of competing interest

The authors declare that they have no known competing financial interests or personal relationships that could have appeared to influence the work reported in this paper.
